# A Motorcycle Paramedic Increases the Survival Rate of Patients after OHCA

**DOI:** 10.3390/medicina59101708

**Published:** 2023-09-24

**Authors:** Mateja Škufca Sterle, Matej Podbregar

**Affiliations:** 1 Faculty of Medicine, University of Ljubljana, 1000 Ljubljana, Slovenia; matej.podbregar@mf.uni-lj.si; 2 Emergency Care Department, Community Health Center Ljubljana, 1000 Ljubljana, Slovenia; 3 Department of Intensive Care, General Hospital Celje, 3000 Celje, Slovenia

**Keywords:** emergency medical services, prehospital emergency care, cardiac arrest, cardiopulmonary resuscitation, motorcycles, response time

## Abstract

*Background and Objectives:* Despite advancements in modern medicine, the survival rate of patients after out-of-hospital cardiac arrest (OHCA) remains low. The proportion of OHCA patients who could be saved under ideal circumstances is unknown. A significant portion of patients experience cardiac arrest due to irreversible conditions. The survival of patients with reversible causes depends on the prompt initiation of basic life support (BLS) and early defibrillation. In order to increase the chances of survival, the motorcycle paramedic (MP) project was implemented in Ljubljana in 2003. The MP is equipped with an AED. In the case of OHCA with a shockable rhythm, he performs defibrillation before the arrival of the emergency medical team (EMT). The aim of this study was to evaluate whether the MP, by reducing response times to OHCA patients, increases the survival and outcome of these patients compared to the EMT. *Materials and Methods:* A retrospective analysis of OHCA cases within the area covered by Ljubljana Emergency Medical Service (EMS) was conducted for the period from January 2003 to December 2022. Instances where the MP arrived at the scene before the EMT were considered MP interventions and classified as the MP group; all other interventions were classified as the EMT group. *Results:* Between January 2003 and December 2022, the EMT performed resuscitation on 3352 patients. In 316 cases, the MP was simultaneously activated and arrived at the scene before the EMT. The response time in the MP group was shorter compared to the EMT group (7.7 ± 4.1 min vs. 9.9 ± 6.5 min, *p* < 0.001). In 16 patients, return of spontaneous circulation (ROSC) was achieved before the arrival of the EMT. The MP group had a higher ROSC rate, a larger proportion of patients were discharged from the hospital and there were more patients with a good neurological outcome compared to the EMT group (44.3% vs. 36.9%, *p* = 0.009; 18.7% vs. 13.0%, *p* = 0.005; 15.9% vs. 10.6%, *p* = 0.004, respectively). *Conclusion:* This study has demonstrated that the implementation of the MP into the EMS in Ljubljana has resulted in shorter response times, an increased survival rate and improved neurological outcome for OHCA patients.

## 1. Introduction

Despite the progress of modern medicine, survival rates after out-of-hospital cardiac arrest (OHCA) remain low both in Slovenia and around the world [1,2,3]. The proportion of OHCA patients who could be saved under ideal circumstances is unknown. A significant portion of patients experience OHCA due to conditions that are irreversible. The fate of patients with reversible causes of cardiac arrest depends on the timely initiation of resuscitation procedures. Numerous studies have demonstrated that only two factors influence the survival of OHCA patients: the immediate or as rapid as possible initiation of basic life support (BLS) procedures and early defibrillation [4]. It has been found that OHCA patients have a threefold higher chance of survival with a good neurological outcome if bystanders perform bystander BLS [5]. The same applies to the early use of AEDs, of which the utilization in Europe remains low [5].

In Europe, the European OHCA Registry has been established over the last 10 years. The first prospective, multicenter study (EuReCa ONE) collected and analyzed data for one month in 2014 [6]. Besides 26 other European countries, Slovenia was also included. It was found that more than half of the OHCA patients received resuscitation, and 10% of resuscitated patients survived for 30 days [6]. This was followed by the EuReCa TWO study, which was similarly an international, prospective, multicenter study and collected data on OHCA for three months in 2017 in 29 European countries, including Slovenia [3]. During the observed period, 8% of resuscitated patients survived until hospital discharge (with variations between countries ranging from 0% to 18%). In addition to EuReCa, in recent years, there have been several reports from individual European countries regarding the survival of OHCA patients, where the numbers also vary significantly: Norway 14% [7], Sweden 11.2% [8], Ireland 6% [8], France 4.9% [9] and Spain 13% [10].

Regarding survival after OHCA in the Ljubljana area, several studies have been published. In the period 1995–1997, 5.3% of patients survived until hospital discharge (3.5% had a good neurological outcome) [11]. In the period 1999–2000, the survival of OHCA patients was 13.6% (with a good neurological outcome of 6.7%) [12]. In the period 2001–2009, the survival rate was 12.4% (with a good neurological outcome of 8.5%) [2], and in the period 2008–2012, it was 13.4% (with a good neurological outcome of 9.3%) [13]. The latest study covered the period 2016–2018, during which the survival rate of OHCA patients was 13.1% (with 10.9% having a good neurological outcome) [1].

In Slovenia, a single-tier emergency medical service (EMS) system is established, meaning that all emergency medical teams (EMTs) always depart from its base, such as the Emergency Department at the University Medical Centre in Ljubljana. The EMS in Ljubljana primarily covers the City Municipality of Ljubljana and an additional nine neighboring municipalities. In addition to its designated area, the Ljubljana EMS also provides secondary coverage for the areas of neighboring EMSs when they are occupied. Therefore, the overall coverage area is quite extensive, and the average response times for the highest-priority cases to the most distant areas can be 30 min or more.

In order to increase the chances of survival, the motorcycle paramedic (MP) project was implemented in Ljubljana in 2003 with the aim of reducing response times. Although MP vehicles lack patient transport capabilities, its shape, size and speed can shorten response times to the patient, which is crucial for a patient in cardiac arrest. Experiences from abroad have shown that the response and access times of MPs are shorter than those of ambulance teams, especially in urban centers [14,15,16,17]. Shorter response times have been proven to be associated with higher patient survival rates and improved neurological outcomes after OHCA [17,18]. 

The work of the MP has some significant limitations—unfortunately, it is restricted to daylight hours from May to November and good weather conditions. Moreover, due to their nature, motor vehicles are far less crashworthy than enclosed vehicles. They are also less visible to other drivers and pedestrians and are less stable than four-wheeled vehicles [19,20]. Motorcyclists are more vulnerable to road conditions and weather hazards compared to enclosed vehicle drivers [21]. No one can predict all the hazards MP might encounter. Therefore, questions and dilemmas often arise, such as: Is it safe enough to include MPs in the EMS system? Does the benefit justify the risk?

The purpose of this study is to determine whether the MP by reducing response times to OHCA patients increases the survival and neurological outcome of these patients.

## 2. Materials and Methods

Study design: A retrospective analysis of cardiac arrests was conducted in the area primarily covered by the Ljubljana EMS for the period from 1 January 2003 to 31 December 2022. This study was observational and causal–comparative in nature. The research protocol received approval by Slovenian Medical Ethics Committee (no. 0120-228/2023/5).

Study setting: The EMS in Ljubljana is centralized and single-tiered, with the EMTs always operating from the base (Emergency Department Ljubljana). The area primarily covered by the EMS of Ljubljana includes not only the City Municipality of Ljubljana but also nine neighboring municipalities. The area spans 904 km^2^ and has a population of 365,000 residents, with the majority residing in the City Municipality of Ljubljana (293,000). On working days, the population increases by approximately 150,000 due to daily migrations to the City Municipality of Ljubljana. In addition to the area primarily covered, the Ljubljana EMS also provides secondary coverage for the areas of neighboring EMS when they are occupied. This secondary coverage area spans 806 km^2^ and has a population of 79,000. Until 2020, a doctor accompanied the ambulance, but in 2020, the practice changed, and the doctor was no longer part of the ambulance crew. Instead, the doctor now arrives at the scene simultaneously with a dedicated vehicle (urgent doctor’s vehicle).

**Figure 1:**
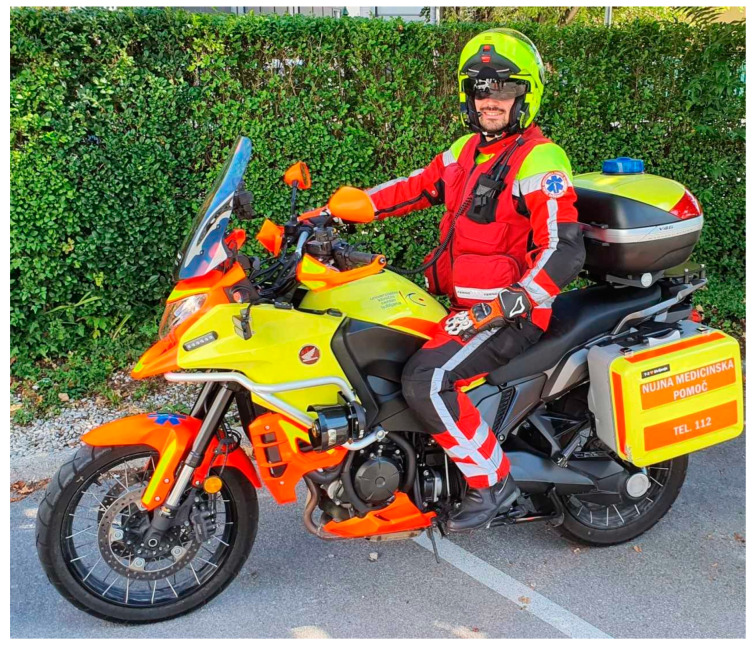
Motorcycle paramedic in Ljubljana (Source: Ambulance Service Ljubljana).

Motorcycle paramedic: A MP has been part of the EMS since 1 June 2003. He is available from May to the end of October during daylight hours and favorable weather conditions. The rescue motorcycle used in Ljubljana is a modified commercial motorcycle (see Figure 1). It is equipped with three blue strobe lights and speakers for emitting audible warning tones. There are three compartments on the motorcycle for storing medical equipment. A stationary VHF radio station, mounted on the handlebars of the motorcycle, is used for communication with the dispatch center. Additionally, a microphone system is integrated into the protective motorcycle helmet for communication during rides [19].

The MP is activated by the dispatch center in all cases of vital threats (e.g., unconsciousness, severe bleeding, etc.), including OHCA or suspicion of it. In addition to other equipment, the MP carries a semi-automatic defibrillator (AED). In the case of OHCA, he initiates BLS procedures and, when dealing with a shockable rhythm, performs early defibrillation—usually even before the arrival of the EMT.

Study population: This study included all patients who underwent resuscitation due to OHCA by EMS Ljubljana from 1 January 2003 to 31 December 2022. This study considered interventions only when the MP and EMT were simultaneously activated. Interventions, when the MP arrived at the scene before the EMT, were categorized as MP interventions and patients were classified as part of the MP group. When the MP arrived at the patient simultaneously or later than EMT, the patients were considered as part of the EMT group. 

Data collection: This study utilized health data obtained from records of urgent interventions at the Health Center Ljubljana, Ambulance Service Ljubljana, NMP3000Web Dispatch Center of Health Ljubljana, records from the urgent care clinics at the University Medical Centre Ljubljana and discharge summaries from hospital departments where treated patients were admitted. Patients for whom health documentation could not be obtained were excluded from this study.

Primary outcome: The primary aim of this study was to compare response times between the MP and the EMT group. 

Secondary outcome: The secondary aim was to assess the impact of MP interventions on the survival and neurological outcomes of OHCA patients.

Power analysis: We assumed a 2-min decrease in response time for the MP group based on published data, which suggests a potential 20% increase in the survival rate [20]. For the estimated error (Type I. error of 0.05, Type II. error of 0.20) and the absolute difference of 2 min in response time (7 ± 5 min in the MP group and 9 ± 5 min in the EMT group, with a sample ratio MP group/EMT group = 0.1), we determined that a sample size of 54 patients in the MP group and 539 patients in the EMT group was necessary. A free online power calculator was used (http://powerandsamplesize.com/Calculators/Compare-2-Means/2-Sample-Equality, accessed on 14 September 2023).

Statistical analysis: The study population was divided into two groups: the MP group and the EMT group. Continuous data were summarized as the mean (±SD) and compared using Student’s *t*-test for independent samples. Non-continuous data were presented as counts (percentages), and the Chi-square test was used to compare non-continuous data. The statistical analysis was performed using IBM SPSS Statistics, version 29.0.0.0 (IBM Corporation, Armonnk, NY, USA). A significance level of *p* < 0.05 was considered statistically significant. 

## 3. Results

Between 1 January 2003 and 31 December 2022, EMS Ljubljana resuscitated a total of 3352 OHCA patients. The MP was present at the scene in 355 cases (10.6% of all resuscitations), with 316 cases (9.4% of all resuscitations) where the MP arrived before the EMT.

Table 1 and Table 2 present the characteristics of both groups. The groups did not significantly differ in terms of average age, cause of cardiac arrest and the performance of BLS procedures before the arrival of the MP and/or the EMT (as shown in Table 1 and Table 2). Both groups were predominantly male, with a slightly higher proportion of males in the MP group (76.9% vs. 69.8%, *p* = 0.008). The majority of cardiac arrests occurred in a home setting, with a lower percentage in the MP group compared to the EMT group (53.0% vs. 63.1%, *p* < 0.001) (as shown in Table 1). In the EMT group, there were more witnesses to the cardiac arrest compared to the MP group (77.6% vs. 71.2, *p* = 0.012) (as shown in Table 2).

The response time in the MP group was shorter compared to the EMT group (7.7 ± 4.1 min vs. 9.9 ± 6.5 min, *p* < 0.001) (Table 3). The proportion of patients with shockable rhythm (i.e., VT/VF) was significantly higher in the MP group compared to the EMT group (36.5% vs. 26.8%, *p* < 0.001) (Table 3).

In the MP group, ROSC was achieved in 16 patients before the arrival of the EMT; this represents 5.1% (16/316) of MP interventions. 

The MP group had a higher rate of ROSC, a larger proportion of patients were discharged from the hospital and there were more patients with a good neurological outcome (cerebral performance category, CPC 1 or 2) compared to the EMT group (44.3% vs. 36.9%, *p* = 0.009; 18.7% vs. 13.0%, *p* = 0.005; 15.9% vs. 10.6%, *p* = 0.004, respectively) (Table 4).

## 4. Discussion

This study has demonstrated that the implementation of the MP into the EMS in Ljubljana has resulted in shorter response times, increased survival rate and improved neurological outcome of OHCA patients.

This study revealed that the average response time of the MP from the call to patient arrival in cases of sudden cardiac arrest is significantly shorter than that of a conventional ambulance vehicle. This can be attributed to the MP’s smaller size, shape and greater speed, which make it much faster, especially in crowded urban areas. These findings are consistent with data in the literature [14,15,16]. Consequently, the proportion of patients with a shockable rhythm on the monitor upon the MP’s arrival is higher compared to the other group. In fact, in sixteen patients in our study, the MP achieved ROSC with early defibrillation before the arrival of the EMT.

Despite the fact that the group where the MP was on-site before the EMT had a significantly lower proportion of patients with witnesses present at the onset of sudden cardiac arrest, the survival of patients in this group was statistically significantly higher. The proportion of patients achieving ROSC, the proportion of patients discharged from the hospital and the proportion of patients with favorable neurological outcomes (CPC 1 or 2) were all significantly higher in the MP group.

The reasons for sudden cardiac arrest are highly diverse. A significant portion of patients experience sudden cardiac arrest due to irreversible causes, which can be acute or a terminal phase of a chronic disease. In such cases, on-site resuscitation cannot be successful. In contrast, patients who suffer sudden cardiac arrest due to reversible causes can benefit from resuscitation procedures. The patient’s outcome can be favorable if resuscitation procedures are initiated immediately or as quickly as possible and defibrillation is performed promptly in the case of shockable rhythms [4]. Studies have shown that a patient with OHCA has three times the greater chances of survival with a favorable neurological outcome if bystanders perform BLS procedures [5]. Similarly, early use of an AED has a positive impact, yet its utilization remains relatively low in Slovenia and Europe [5]. Numerous studies have demonstrated that two factors have the most significant influence on OHCA patient survival: immediate or as quick as possible initiation of BLS procedures and early defibrillation of VF/VT rhythms [4].

Despite the progress of modern medicine, survival after OHCA remains low [1,2,3]. Part of the reason lies in the fact that a significant portion of patients suffer from irreversible conditions where resuscitation efforts cannot be successful. The cause of cardiac arrest is often challenging for the on-site EMT to identify during resuscitation, as diagnostic capabilities in the field, despite considerable medical advancements, are still quite limited. The EMT frequently lacks even basic information about the patient’s general condition and medical history at the beginning of resuscitation. Another reason for the low survival rate after OHCA is the delay in initiating resuscitation procedures (long response times). The longer the response times and delays in starting resuscitation, the lower the chances of patient survival [17,18].

In Ljubljana, a one-tier EMS system has been in place for several years, which is incapable of saving most patients with reversible causes of sudden cardiac arrest unless the arrest happens in the presence of the EMT or in its immediate vicinity. The one-tier system of EMS is a centrally oriented organization where all teams are concentrated and dispatched from the base in Ljubljana. A weakness of such an organization lies is the long and unevenly distributed response times of the EMT [22].

**Figure 2:**
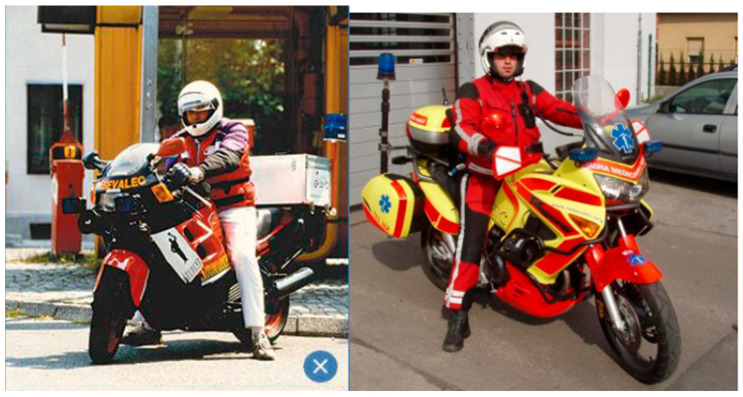
Motorcycle paramedic in the past (Robert Sabol) on the left picture and today on the right picture [19].

With the aim of reducing response times to patients with OHCA in the existing EMS in Ljubljana, the MP project was introduced in 2003. The idea was not new, as its initial roots can be traced back to 1956 when a neighboring Austrian automobile club introduced roadside assistance using a motorcycle equipped with slightly more advanced equipment for providing aid [19]. The use of rescue motorcycles in the EMS was already known in the second half of the 20th century in several European countries as well as in the USA [19,23]. The Chicago Fire Department, inspired by motorcycle police officers, introduced motorcycle paramedics into their organization in 1979 [23]. The idea was not new in our context either; as in 1994, inspired by successful experiences abroad and on the initiative of a businessperson in Ljubljana, attempts were made to introduce a motorcycle paramedic into the EMS in Ljubljana [19]. The motorcycle was provided by the aforementioned businessperson, but it was not as suitable for such activities as today’s motorcycles (Figure 2) [19]. The MP did not use full protective gear and did not have an AED among his equipment.

The first MP in Ljubljana and Slovenia was paramedic Robert Sabol (Figure 2), who worked as a MP in his free time for three months in 1994. He carried out 26 interventions, which was insufficient to gain approval from the Ministry of Health for the project [19]. However, in 2003, the MP was formally introduced into the EMS system in Ljubljana and continues to operate to this day [19]. 

MPs in Ljubljana are employed at the Ljubljana Ambulance Service. To work as a MP, individuals must meet specific requirements, including education and work experience as a paramedic, a valid motorcycle license and experience in motorcycle riding. Prior to the start of each season, rigorous preparations are undertaken, including safe driving training and physical and psychological preparation [19].

This study demonstrated that the implementation of the MP significantly increased patient survival, despite the fact that the MP was relatively rarely (9.4% of cases) on-site before the arrival of the EMS team. There are numerous limitations to the MP’s capabilities. For safety reasons, the MP is available only from the beginning of May to the end of October, in favorable weather conditions, during daylight hours and when not occupied with another intervention. Consequently, only a relatively small number of OHCA patients benefit from their services. This study also revealed that the MP was activated later than the ambulance vehicle for thirty-nine OHCA patients, even though they were available (11% of all motorcycle paramedic activations).

This study also has limitations. We compared the outcomes of those patients with OHCA where the MP was on-site before the EMT. However, MPs are available only during specific seasons, during the day, and in good weather. Ideally, the control group would also have the same limitations, but for ethical reasons, this is not possible.

To ensure a reduction of response times for as many OHCA patients as possible in Slovenia, it would be necessary to introduce a two-tier EMS system alongside the optimization of activating the MP. In a two-tier EMS system, emergency ambulances and MPs are strategically located across the region based on probabilistic models of emergency incidents. In the case of life-threatening patients, such as OHCA, the nearest ambulance or MP would be dispatched first, arriving in a shorter response time. This approach, combined with the establishment of an effective system of certified first responders, an adequate number of publicly accessible AEDs and the education and encouragement of bystanders by dispatchers, can significantly reduce the response times for initiating of resuscitation and early defibrillation in cases of shockable rhythms. Such measures could result in improved chances of survival for those patients who experience sudden cardiac arrest due to reversible causes in the future.

In the 20 years of MP experiences in Ljubljana, with an average of 500 interventions carried out by MPs in each season, there have been no accidents involving MPs resulting in their death or the death of anyone else involved in the accident. There was one traffic accident that resulted in disability for the MP. Two other accidents resulted in severe injuries, but the MPs were able to return to work. Additionally, there were some slips and minor accidents without injuries or with minor injuries to the motorcycle rescuer.

Based on the number of interventions conducted, the incidence of traffic accidents involving MPs in Ljubljana is very low. The research results support the standpoint that the MPs play an important role in the EMS. With the appropriate selection of experienced motorcyclists, specific defensive driving training and proper protective equipment, we can minimize the number of accidents and their consequences.

## 5. Conclusions

The response time to a patient with OHCA and the initiation of resuscitation procedures are of vital importance. The implementation of the MP in the EMS system in Ljubljana has significantly shortened the response times to these patients. This study has also demonstrated that the implementation of the MP into the EMS system in Ljubljana has increased the survival rate and neurologic outcome of OHCA patients despite all limitations. 

## Figures and Tables

**Table 1:** Basic characteristics of patients in both groups.

	MP Group	EMT Group	Statistics *p*
Age (years)	63.4 ± 18.2	64.8 ± 17.0	0.170
Male, *n* (%)	243 (76.9%)	2118 (69.8%)	0.008
Location of OHCA: Domestic environment, *n* (%)	167 (53.0%)	1907 (63.1%)	<0.001
Non-traumatic cause of OHCA, *n* (%)	298 (94.3%)	2902 (95.6%)	0.297

OHCA: out-of-hospital cardiac arrest, MP: motorcycle paramedic, EMT: emergency medical team.

**Table 2:** Presence of witnesses of the cardiac arrest and the performance of BLS procedures before the arrival of the motorcycle paramedic/EMT.

	MP Group	EMT Group	Statistics *p*
Bystanders present, *n* (%)	223 (71.2%)	2064 (77.6%)	0.012
Performing of BLS, *n* (%)	191 (61.8%)	1642 (62.1%)	0.927

BLS: basic life support, MP: motorcycle paramedic, EMT: emergency medical team.

**Table 3:** Response times and initial rhythm on the monitor at the beginning of resuscitation.

	MP Group	EMT Group	Statistics *p*
Response time (min)	7.70 ± 4.1	9.93 ± 6.5	<0.001
Initial rhythm VF/VT, *n* (%)	115 (36.5%)	813 (26.8%)	<0.001

Response time: time from call to patient arrival, VF/VT: ventricular fibrillation/ventricular tachycardia, MP: motorcycle paramedic, EMT: emergency medical team.

**Table 4:** OHCA outcomes.

	MP Group	EMT Group	Statistics *p*
ROSC, *n* (%)	140 (44.3%)	1119 (36.9%)	0.009
Discharged from the hospital, *n* (%)	59 (18.7%)	394 (13.0%)	0.005
CPC 1 or 2 at discharge, *n* (%)	50 (15.9%)	321 (10.6%)	0.004

OHCA: out-of-hospital cardiac arrest, ROSC: return of spontaneous circulation, CPC: cerebral performance category, MP: motorcycle paramedic, EMT: emergency medical team.

## Data Availability

The database is available through the first author.
